# Perceived Helicopter Parenting and Learning Burnout in Chinese Adolescents: Intelligence Mindsets and Psychological Capital as Mediators

**DOI:** 10.3390/bs16081351

**Published:** 2026-08-06

**Authors:** Yongqiang Jiang, Lina Feng, Huixiao Le

**Affiliations:** 1 Faculty of Education, Beijing Normal University, Beijing 100875, China; jiangqq1991@bnu.edu.cn; 2 Beijing Institute of Educational Sciences, Beijing 100036, China; linaa10@163.com; 3 Shanghai Institute of AI for Education, Eastern China Normal University, Shanghai 200062, China

**Keywords:** helicopter parenting, learning burnout, fixed mindset, psychological capital, mediation model

## Abstract

This study aimed to examine the association between perceived helicopter parenting and adolescents’ learning burnout, as well as the potential mediating roles of intelligence mindsets and psychological capital. A sample of 3392 Chinese adolescents (1635 7th-graders and 1757 8th-graders) from three junior high schools completed self-report measures of helicopter parenting, fixed and malleable intelligence mindsets, psychological capital, and learning burnout. Structural equation modeling showed that perceived helicopter parenting was positively associated with learning burnout, both directly and indirectly. Significant indirect associations were through fixed mindset, psychological capital, and their sequential pathway, whereas pathways involving malleable mindset were not significant. Multigroup analyses further revealed several pathway-specific gender differences. These findings highlight the importance of distinguishing fixed and malleable mindsets and suggest that psychological capital may be a central proximal resource linking perceived helicopter parenting with adolescents’ learning burnout.

## 1. Introduction

Children’s academic achievement is not merely a personal milestone but a significant source of familial expectancy and pride (Chao & Sue, 1996). This issue becomes particularly salient during junior high school, when students face increasing academic demands and prepare for the competitive transition to senior high school. Although the 2021 “Double Reduction” policy was intended to ease students’ academic burden, recent evidence suggests that parental educational anxiety and academic involvement remain substantial in many families (Z. Qi et al., 2024; X. Zhu & Luo, 2023). When parental involvement becomes excessive, overprotective, and intrusive, it may take the form of helicopter parenting. Such parenting may be associated with adolescents’ learning burnout, especially when academic support is experienced as pressure or control rather than autonomy-supportive involvement. This study therefore aimed to examine the association between adolescents’ perceived helicopter parenting and learning burnout among Chinese junior high school students and further explore the underlying psychological mechanisms.

### 1.1. Helicopter Parenting

Helicopter parenting, often discussed under the broader label of overparenting, refers to a developmentally inappropriate form of parental involvement characterized by excessive monitoring, overprotection, and intervention in matters that young people could increasingly manage on their own (Padilla-Walker & Nelson, 2012). It is related to, but conceptually distinct from, several adjacent constructs. Parental control is a broader construct that may include both adaptive behavioral regulation and maladaptive control, depending on its form and developmental appropriateness. Psychological control refers more specifically to manipulative practices that intrude into the child’s psychological and emotional world, such as guilt induction or love withdrawal. Academic involvement refers to parents’ participation in children’s learning and schooling and can be beneficial when it is autonomy-supportive. By contrast, helicopter parenting emphasizes the excessive and intrusive quality of involvement, especially when parents overmanage decisions, closely monitor activities, or intervene in problems that adolescents should be learning to handle independently. In this sense, helicopter parenting is not simply “high involvement,” but involvement that is experienced as developmentally excessive and autonomy-limiting. Previous studies have linked helicopter parenting to poorer adjustment among youth, including lower autonomy, emotional difficulties, and academic maladjustment (Ching et al., 2023b; Gao et al., 2023; L. Li et al., 2024; W. Wu et al., 2023).

Over the past two decades, research on helicopter parenting has predominantly focused on its impact on emerging adults (Cui et al., 2022; La Rosa et al., 2025). It is also relevant to junior high school students. Early to middle adolescence is marked by increasing needs for autonomy, self-direction, and independent problem-solving, while parents may continue to closely supervise adolescents’ academic and daily activities (Aquilino, 1997). During this formative phase, the adverse effects of helicopter parenting may also be pronounced. Recognizing this, a growing body of research has begun to extend the investigation of helicopter parenting to the younger population (e.g., Ching et al., 2023b; Leung, 2020; Pistella et al., 2022; Y. Wang et al., 2025a). This tension may be particularly salient in China, where parental academic involvement is culturally valued and closely tied to educational competition, family expectations, and the notion of training (管) (Chao & Sue, 1996; Zong & Hawk, 2022). Importantly, culturally normative academic involvement should not be equated with helicopter parenting. Rather, such involvement may become helicopter parenting when adolescents perceive it as excessive, intrusive, or controlling. Parents frequently view their high involvement as appropriate care, nurturance, and emotional support, whereas adolescents often perceive the same actions as intrusive, overbearing, and psychologically controlling (Y. Wang et al., 2025c). Consequently, to accurately capture the sense of intrusiveness actually experienced by youth, the current study focuses specifically on adolescents’ perceived helicopter parenting.

### 1.2. Helicopter Parenting and Learning Burnout

Learning burnout, also referred to as academic burnout, is typically characterized by academic exhaustion, cynicism toward schoolwork, and a diminished sense of accomplishment (Salmela-Aro & Upadyaya, 2014). Among Chinese junior high school students, learning burnout is highly prevalent and has meaningful implications for school adjustment and broader psychological functioning (Q. Hu & Dai, 2007; Z. Liang & Shao, 2019). If left unaddressed during early adolescence, learning burnout may compromise both academic functioning and personal well-being and may even contribute to more severe outcomes such as school disengagement or refusal (L. Liu et al., 2021). Although the etiology of learning burnout is multifaceted (Z. C. Liang et al., 2025), the present study focuses on perceived helicopter parenting as one salient family-level correlate of burnout. This focus is especially relevant in contexts where parental involvement in academic matters is intensive and adolescents may experience such involvement as pressure or control.

Self-determination theory provides a useful framework for understanding why perceived helicopter parenting may be associated with learning burnout. According to this theory, adolescents’ motivation and well-being depend partly on the satisfaction of basic psychological needs, especially autonomy and competence (Ryan & Deci, 2000). When parents closely monitor schoolwork, overmanage academic decisions, or intervene in problems that adolescents could increasingly handle independently, adolescents may experience learning as externally controlled rather than self-endorsed. Such experiences may be associated with lower intrinsic motivation, weaker perceived competence, and greater emotional exhaustion or cynicism toward schoolwork. Consistent with this reasoning, empirical studies have demonstrated that helicopter parenting is associated with maladaptive academic motivation (Schiffrin & Liss, 2017), lower school engagement (Padilla-Walker & Nelson, 2012), and higher levels of school-related burnout (Cengiz & Peker, 2024; Ching et al., 2023b; Love et al., 2020).

### 1.3. Intelligence Mindsets and Psychological Capital as Potential Mediators

When parents overmanage academic and daily challenges, adolescents may have fewer opportunities to experience self-directed effort, mastery, and problem-solving. Such experiences are closely related to how adolescents understand ability and how they develop positive psychological resources for coping with academic demands. Accordingly, we considered intelligence mindsets as cognitive-motivational beliefs and psychological capital as a more proximal psychological resource that may be associated with learning burnout.

#### 1.3.1. Intelligence Mindsets

Intelligence mindsets, or implicit theories of intelligence, refer to an individual’s core beliefs about whether intellectual ability is fixed or malleable (Yeager & Dweck, 2012). A malleable mindset reflects the belief that intelligence can be developed through effort, strategies, and learning, whereas a fixed mindset reflects the belief that intelligence is relatively stable. These beliefs are closely related to students’ responses to academic challenges. A malleable mindset is generally associated with adaptive coping, persistence, and engagement, whereas a fixed mindset is more often associated with helpless responses, avoidance of challenge, and poorer academic adjustment (Claro et al., 2016; Costa & Faria, 2018; Kim & Park, 2021; Rege et al., 2020). A growing body of research specifically links weaker malleability beliefs or stronger fixed beliefs to higher levels of learning burnout and poorer school-related well-being (e.g., Nieuwenhuis et al., 2023; Zhang et al., 2025).

Historically, intelligence mindsets were conceptualized as opposite poles of a single continuum. However, a substantial body of contemporary research supports a two-dimensional model, demonstrating that malleable and fixed mindsets are largely independent and can coexist within the same individual (Costa & Faria, 2018; Lee & Seo, 2019; Yan & Schuetze, 2023). The distinction is also reflected in their underlying cognitive processes: a malleable mindset is often associated with self-referenced mastery goals and learning from mistakes, while a fixed mindset is linked to other-referenced performance goals and social comparison (Chiu et al., 2023). This duality is particularly salient in Chinese culture, where proverbs like “diligence can compensate for dullness (勤能补拙)” simultaneously acknowledge the value of effort (aligned with a malleable mindset) and imply an inherent starting point (resonant with a fixed mindset). Recent longitudinal evidence from Chinese adolescents shows that individuals high in both mindsets experience the poorest academic outcomes (C. Qi et al., 2025). This finding highlights the complex interplay between these beliefs and underscores the necessity of examining them as separate, co-occurring dimensions.

Adolescence is a formative period for the development of intelligence mindsets and related motivational characteristics (Park et al., 2020), yet their antecedents, particularly within the family context, warrant further investigation. An autonomy-supportive environment, characteristic of authoritative parenting, fosters a malleable mindset by encouraging self-initiation and agency (Ren et al., 2025). By contrast, helicopter parenting may be less conducive to adaptive mindset development because it limits adolescents’ opportunities to experience success and failure as outcomes of their own effort, strategies, and problem-solving. Its intrusive and overcontrolling nature may also heighten fear of negative evaluation (Carr et al., 2021), thereby making adolescents more likely to view ability as something that is constantly judged and relatively fixed. Consistent with this reasoning, prior research has linked helicopter parenting to a stronger fixed mindset (Ching et al., 2023a). However, less is known about whether helicopter parenting is also related to adolescents’ malleable mindset. Therefore, the present study examined fixed and malleable mindsets separately as potential mediators, allowing us to evaluate whether perceived helicopter parenting is associated with learning burnout through distinct mindset-related pathways.

#### 1.3.2. Psychological Capital

Psychological capital refers to a positive, state-like psychological resource consisting of self-efficacy, hope, resilience, and optimism (Luthans & Youssef-Morgan, 2017). In academic settings, these resources may help adolescents believe in their capacity to handle schoolwork, pursue academic goals, recover from setbacks, and maintain positive expectations. From a resource perspective, learning burnout is more likely when students lack sufficient psychological resources to cope with sustained academic demands. Consistent with this view, psychological capital has been associated with greater academic engagement, better perceived achievement, and higher student well-being (e.g., N. Chen et al., 2025; Y. Hu et al., 2025; Song et al., 2025; H. Wang et al., 2023).

Psychological capital is an individual’s malleable positive psychological development state and can be cultivated and strengthened through social mechanisms, such as constructive relationships and positive family interactions (Youssef-Morgan & Luthans, 2015). Empirical research confirms that family factors, like the quality of parent–child relationships and family support, are significant antecedents of adolescents’ psychological capital (X. Liu et al., 2024; H. Wang et al., 2023; You et al., 2025). Helicopter parenting, despite its intensive involvement, typically lacks genuine autonomy support. Its intrusive and controlling features may be associated with fewer opportunities for adolescents to practice autonomous coping, solve manageable problems independently, and develop confidence in handling academic challenges. Therefore, psychological capital may serve as a proximal psychological resource linking perceived helicopter parenting with adolescents’ learning burnout.

#### 1.3.3. Intelligence Mindsets and Psychological Capital

The link between intelligence mindsets and psychological capital may be understood as a theoretically plausible cognitive-to-resource pathway. Intelligence mindsets represent adolescents’ basic beliefs about ability and are closely tied to how they interpret effort, difficulty, and failure (Yeager & Dweck, 2012). Adolescents who believe that ability can be developed may be more likely to view setbacks as improvable, sustain hope and optimism, and maintain confidence and resilience when facing academic challenges. In contrast, those who view ability as fixed may be more likely to interpret difficulties as evidence of limited competence, which may weaken their positive psychological resources. Consistent with this reasoning, prior studies have treated growth mindset as an antecedent of psychological capital and found positive associations between growth mindset and adolescents’ psychological capital (Zhang et al., 2023; Zhao et al., 2023). Therefore, the present study specified intelligence mindsets as cognitive-motivational factors that may be associated with psychological capital, which in turn may be more proximally related to learning burnout.

### 1.4. Adolescents’ Gender as a Potential Moderator

Adolescents’ gender may serve as an important moderator. From the perspective of gender socialization and social role theory, boys and girls are embedded in different societal expectations, which may shape how they interpret parental involvement, respond to academic demands, and mobilize psychological resources (Eagly et al., 2000; Hill & Lynch, 1983). Previous studies have reported gender differences in the associations between parental control or involvement and adolescents’ psychological adjustment (Bacikova-Sleskova et al., 2024), as well as in the development of school burnout (Salmela-Aro & Tynkkynen, 2012). In Chinese youth, parental involvement and parent–child relational processes have been linked to subjective well-being and psychological capital in gender-differentiated ways (S. Li et al., 2024; You et al., 2025). In addition, recent evidence suggests that parental autonomy support and psychological control are differentially associated with malleable mindset and fixed mindset, with these pathways varying by gender (S. Chen et al., 2025). Taken together, the present study further examined whether the proposed associations among perceived helicopter parenting, intelligence mindsets, psychological capital, and learning burnout differed between boys and girls.

### 1.5. The Present Study

The present study aimed to examine the association between perceived helicopter parenting and adolescents’ learning burnout among Chinese junior high school students, as well as the potential mediating roles of intelligence mindsets and psychological capital. Based on the literature reviewed above, the present study addressed the following hypotheses:

**H1.**  *Perceived helicopter parenting would be positively associated with adolescents’ learning burnout.*

**H2.**  *Perceived helicopter parenting would be negatively associated with psychological capital, whereas psychological capital would be negatively associated with learning burnout.*

**H3.**  *Intelligence mindsets and psychological capital would mediate the association between perceived helicopter parenting and learning burnout. Specifically, perceived helicopter parenting would be associated with lower malleable mindset and higher fixed mindset. Fixed mindset would be positively associated with learning burnout, whereas malleable mindset would be negatively associated with learning burnout.*

**H4.**  *Intelligence mindsets and psychological capital would form sequential indirect pathways linking perceived helicopter parenting to learning burnout, such that perceived helicopter parenting would be associated with intelligence mindsets, which would in turn be associated with psychological capital and subsequently learning burnout.*

**H5.**  *The proposed structural paths would differ between boys and girls.*

## 2. Methods

### 2.1. Participants and Procedure

Participants were 3392 adolescents (1548 girls) recruited from three public junior high schools in county-level areas of Shandong Province, an eastern coastal region of China characterized by a highly competitive educational environment. The participating schools were recruited using a convenience cluster sampling approach based on accessibility and willingness to participate. After obtaining permission from school administrators, students in Grades 7 and 8 were invited to participate as intact groups. The sample consisted of 1635 seventh-grade students and 1757 eighth-grade students. Individual chronological age was not collected in the current study. Participants were enrolled in the first two years of junior high school within the Chinese compulsory education system. Detailed demographic characteristics of the sample are presented in Table 1.

Data were collected through online questionnaires administered in the schools’ computer rooms of the participating schools under standardized instructions provided by trained researchers. Prior to completing the survey, students were informed about the purpose, procedures, voluntary nature, and confidentiality of the study. Written informed consent was obtained from parents or legal guardians, and students provided electronic assent before proceeding with the questionnaire. The study was conducted in accordance with the ethical principles of the Declaration of Helsinki and was reviewed and approved by the Institutional Review Board of first author’s institute.

### 2.2. Measures

Perceived helicopter parenting. Helicopter parenting was measured using the scale developed by Schiffrin et al. (2019). Although this construct has often been examined among older adolescents and emerging adults, parental overinvolvement and autonomy-related parenting practices are also developmentally relevant during junior high school, when adolescents begin to seek greater independence in academic and daily decision-making. Therefore, we adapted the scale for Chinese junior high school students. The scale was translated into Chinese by two bilingual researchers in psychology and education. A different bilingual researcher then back-translated the Chinese version into English. Discrepancies between the original and back-translated versions were discussed and resolved by the research team. To enhance cultural appropriateness for Chinese junior high school students, the translated items were reviewed by experts familiar with adolescent development and school mental health education. This scale originally contains 10 items rated on a 5-point Likert scale ranging from 1 (strongly disagree) to 5 (strongly agree). Preliminary exploratory factor analysis indicated a dominant one-factor structure, explaining 56.61% of the total variance. However, one item (“My parent supervised my every move growing up”) showed a distinct loading pattern and formed a singleton component. This item was therefore removed. The final Chinese version retained nine items. Confirmatory factor analysis further supported the acceptability of the one-factor model, *χ*^2^/*df* = 7.10, *p* < 0.001, CFI = 0.995, TLI = 0.988, RMSEA = 0.042, SRMR = 0.012, with standardized factor loadings ranging from 0.62 to 0.86. In the present study, the Cronbach’s alpha coefficient for the nine-item scale was 0.92.

Intelligence Mindset. Implicit theories of intelligence (intelligence mindset) were assessed using Dweck’s mindset scale (Dweck, 1999). This scale contains eight items that can be categorized into two subscales: four items measuring malleable mindset (e.g., “People can always substantially change how intelligent they are.”) and four items measuring fixed mindset (e.g., “Everyone has a certain amount of ability and they can’t truly do much to change it.”). Participants responded on a 5-point Likert scale ranging from 1 (strongly disagree) to 5 (strongly agree). The scores for malleable and fixed mindsets were calculated by averaging the participants’ responses to the respective four items, with higher scores indicating a greater endorsement of each mindset. In the present study, the Cronbach’s alpha coefficients were 0.77 for malleable mindset and 0.83 for fixed mindset.

Psychological Capital. Psychological capital was measured using the Positive Psychological Capital Questionnaire developed by Zhang et al. (2010). This 26-item scale comprises four dimensions: self-efficacy (7 items), resilience (7 items), hope (6 items), and optimism (6 items). Participants rated each item on a 7-point Likert scale ranging from 1 (strongly disagree) to 7 (strongly agree). Subscale scores were computed by averaging the corresponding items, with higher scores indicating higher levels of each dimension. The Cronbach’s alpha coefficients for self-efficacy, resilience, hope, and optimism were 0.91, 0.79, 0.86, and 0.87, respectively. These four subscale scores were used to construct the latent variable of psychological capital.

Learning Burnout. Learning burnout was assessed using the Adolescent Learning Burnout Scale developed by Y. Wu et al. (2010). This scale consists of 16 items across three dimensions: exhaustion (4 items), academic cynicism (5 items), and low achievement (7 items). All items were rated on a 4-point Likert scale ranging from 1 (completely untrue) to 4 (completely true). Subscale scores were computed by averaging the corresponding items, with higher scores indicating higher levels of each dimension. The Cronbach’s alpha coefficients for exhaustion, academic cynicism, and low achievement were 0.81, 0.76, and 0.84, respectively. These three subscale scores were used to construct the latent variable of learning burnout.

Control Variables. Several demographic and family background variables were controlled for in this study, including grade (7th or 8th), gender, ethnicity (Han vs. ethnic minorities), only-child status, family structure (intact two-parent family vs. others), and subjective socioeconomic status. Subjective socioeconomic status was measured using a single item asking participants to rate their family’s economic condition within the entire school on a 9-point scale ranging from 1 (the lowest level) to 9 (the highest level). These variables were selected because adolescents’ learning burnout, motivational beliefs, and psychological resources may vary by developmental stage and gender, and may also be shaped by family background and socioeconomic conditions (Guo & Li, 2025; Salmela-Aro & Tynkkynen, 2012; Q. Zhu et al., 2023).

### 2.3. Data Analysis

Data analyses were conducted using SPSS 26.0 and Mplus 7.0 (Muthén & Muthén, 1998–2012). Preliminary analyses were first performed to examine potential common method bias and the possible influence of the nested data structure. Harman’s single-factor test was conducted as a preliminary assessment of common method bias (Podsakoff et al., 2003), and intraclass correlation coefficients (ICCs) were estimated using unconditional multilevel models to examine whether school-level clustering should be considered in the subsequent analyses. Descriptive statistics were then calculated for all study variables. Gender differences in the main variables were examined using independent-samples *t* tests, with effect sizes reported as Cohen’s *d*. Pearson correlation analyses were conducted to examine bivariate associations among the main study variables.

Structural equation modeling (SEM) was then conducted. Before testing the hypothesized structural model, the measurement model was examined. Model fit was evaluated using multiple fit indices (L. T. Hu & Bentler, 1999), including the chi-square statistic, comparative fit index (CFI), Tucker–Lewis index (TLI), root mean square error of approximation (RMSEA), and standardized root mean square residual (SRMR). Following commonly used guidelines, CFI and TLI values around or above 0.90 were considered acceptable, and RMSEA and SRMR values around or below 0.08 were considered indicative of acceptable model fit (Kline, 2016). Next, the hypothesized structural model was tested in the total sample. In this model, perceived helicopter parenting was specified as the predictor, fixed mindset, malleable mindset, and psychological capital were specified as mediators, and learning burnout was specified as the outcome variable, while control variables were included as covariates. Statistical significance was evaluated using *p* values and 95% confidence intervals. To examine whether the measurement structure was comparable across gender, multigroup measurement invariance analyses were conducted. Configural, metric, and scalar invariance models were tested sequentially (F. F. Chen, 2007). Changes in model fit were used to evaluate measurement invariance, with changes in CFI, RMSEA, and SRMR considered in addition to chi-square values, given the sensitivity of chi-square tests to large sample sizes. Finally, gender differences in the structural paths were examined using multigroup SEM. Based on the supported measurement invariance results, the measurement parameters were constrained to equality across gender groups, whereas the focal structural paths were freely estimated in the unconstrained model. This model was compared with a constrained model in which the focal structural paths were set to be equal across boys and girls. A significant deterioration in model fit for the constrained model (Δ*χ*^2^) was interpreted as evidence that at least some structural paths differed by gender. Path-specific gender differences were further examined using parameter contrasts. Gender differences in indirect effects were also examined using parameter contrasts of group-specific indirect effects, with 95% confidence intervals reported.

## 3. Results

### 3.1. Preliminary Analyses

Because data were collected from students’ self-reports, Harman’s single-factor test was conducted to assess the potential influence of common method bias. All measurement items were entered into an exploratory factor analysis without rotation. The results indicated that the first factor accounted for 28.79% of the total variance, which was below the conventional threshold of 40%, suggesting that common method bias was unlikely to substantially affect the results. Additionally, because participants were recruited from three schools, preliminary analyses were conducted to examine potential clustering effects. ICCs were estimated for all major study variables using unconditional multilevel models. The ICCs ranged from 0.000 to 0.069, indicating that school-level differences accounted for only a small proportion of the total variance. Detailed information is reported in Supplementary Table S1. Given the limited clustering effects, subsequent analyses were conducted at the individual level.

### 3.2. Descriptive Statistics

Table 2 displays the means and standard deviations of study variables in the total sample and by gender. Skewness and kurtosis values were within acceptable ranges. Independent-samples *t*-tests revealed the significant gender differences, except for perceived helicopter parenting and academic cynicism. Specifically, compared to boys, girls reported significantly higher fixed mindset (*t* = 2.18, *p* = 0.029), lower malleable mindset (*t* = −2.91, *p* = 0.004), lower self-efficacy (*t* = −6.46, *p* < 0.001,), lower resilience (*t* = −8.90, *p* < 0.001), lower hope (*t* = −1.99, *p* = 0.047), and lower optimism (*t* = −3.46, *p* < 0.001), but higher exhaustion (*t* = 4.51, *p* < 0.001) and higher low achievement (*t* = 5.27, *p* < 0.001).

Table 3 shows the correlation coefficients among study variables, exhibiting a similar pattern across gender. Perceived helicopter parenting was not significantly correlated with malleable mindset, but significantly with fixed mindset with a positive valence, indicators of psychological capital with a negative valence, and indicators of learning burnout with a positive valence. The correlations between fixed mindset and malleable mindset across gender were negative and in a low level (*r* = −0.20, *p* < 0.001).

### 3.3. Structural Equation Models

The measurement model showed an acceptable fit to the data, *χ*^2^ = 2161.89, *df* = 88, *p* < 0.001, CFI = 0.940, TLI = 0.918, RMSEA = 0.083, SRMR = 0.072. Although the RMSEA was slightly above the conventional 0.08 threshold, the CFI, TLI, and SRMR values were within acceptable ranges. All the factor loadings were significant (*β*s = 0.54 to 0.86, *p* < 0.001), indicating that the latent variables can be represented by their respective indicators. The standardized factor loadings for the measurement model are reported in Supplementary Table S2.

**Figure 1:**
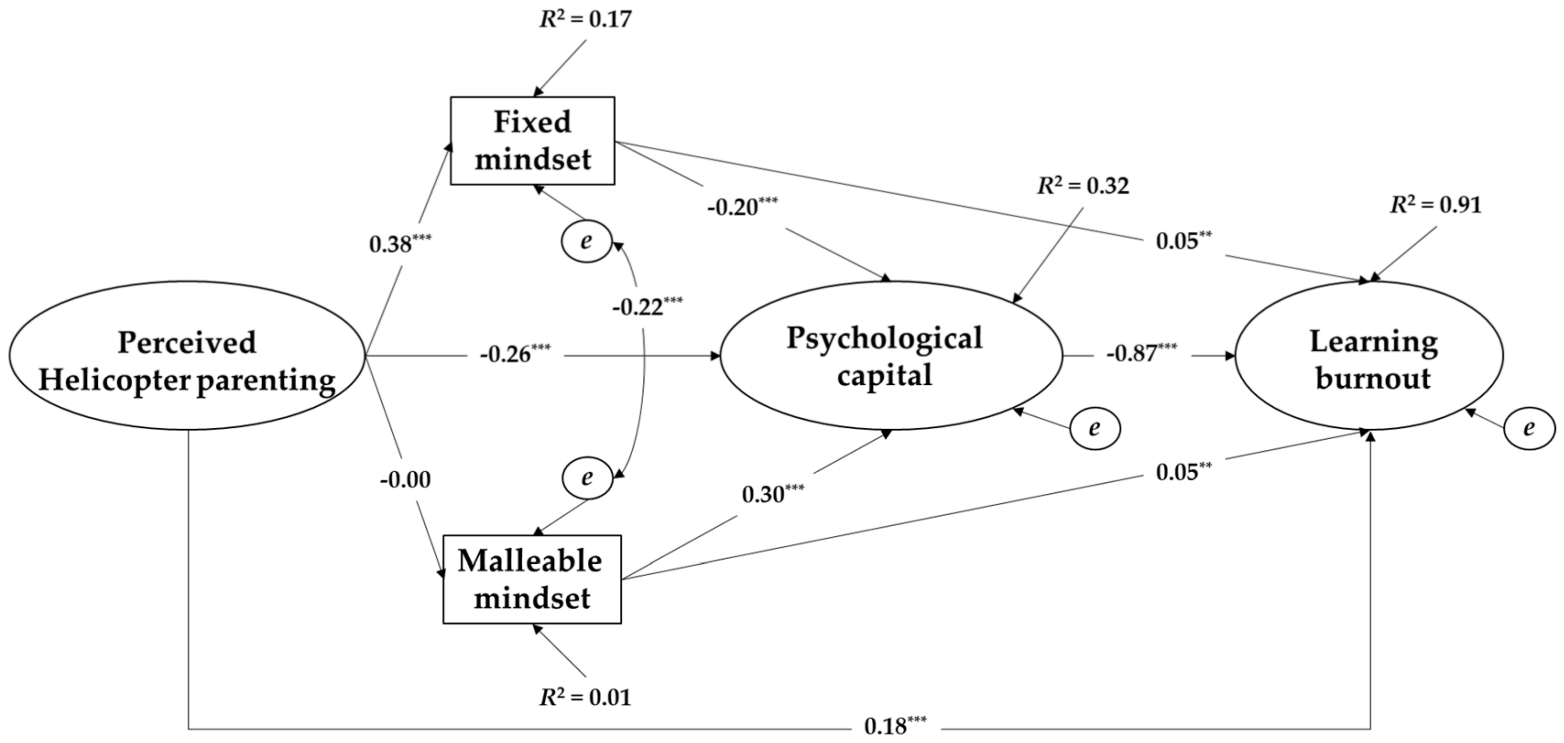
Standardized path coefficients in the Mediation model for the total sample. Note. ** *p* < 0.01, *** *p* < 0.001. Standardized coefficients are presented. Control variables and factor loadings are omitted from the figure for clarity.

Then, the structural model was tested. The model fitted the data well, *χ*^2^ = 2393.45, *df* = 192, *p* < 0.001, CFI = 0.927, TLI = 0.901, RMSEA = 0.058, SRMR = 0.052. As shown in Figure 1, perceived helicopter parenting significantly related to fixed mindset (*β* = 0.38, *p* < 0.001), psychological capital (*β* = −0.26, *p* < 0.001), and learning burnout (*β* = 0.18, *p* < 0.001) but not malleable mindset (*β* = −0.00, *p* = 0.956); fixed mindset (*β* = −0.20, *p* < 0.001) and malleable mindset (*β* = 0.30, *p* < 0.001) were both significantly related to psychological capital, which in turn predicted learning burnout (*β* = −0.87, *p* < 0.001), and the direct prediction from fixed mindset (*β* = 0.05, *p* = 0.002) and malleable mindset (*β* = 0.05, *p* = 0.001) on learning burnout was significant. For the indirect effects, the pathway through fixed mindset directly to learning burnout was significant (*b* = 0.011, *p* = 0.003, 95% CI [0.004, 0.018]), the pathway through psychological capital directly to learning burnout was significant (*b* = 0.119, *p* < 0.001, 95% CI [0.095, 0.142]), and the serial pathway through fixed mindset and psychological capital was significant (*b* = 0.036, *p* < 0.001, 95% CI [0.028, 0.044]). However, the pathway through malleable mindset alone (*b* = 0.000, *p* = 0.956, 95% CI [−0.001, 0.001]) and the serial pathway through malleable mindset and psychological capital (*b* = 0.000, *p* = 0.956, 95% CI [−0.005, 0.006]) were not significant.

Measurement invariance across gender was first examined by testing configural, metric, and scalar invariance models sequentially. The configural model showed an acceptable fit to the data, indicating that the same factor structure was supported for boys and girls (*χ*^2^ = 2259.95, *df* = 176, *p* < 0.001, CFI = 0.939, TLI = 0.917, RMSEA = 0.084, SRMR = 0.074). Constraining factor loadings to be equal across gender did not result in a substantial decrease in model fit (ΔCFI = −0.001, ΔRMSEA = −0.002, ΔSRMR = 0.002), supporting metric invariance. Further constraining item intercepts to equality also resulted in negligible changes in fit indices (ΔCFI = −0.004, ΔRMSEA = −0.001, ΔSRMR = 0.002), supporting scalar invariance. These findings indicate that the latent constructs were comparable across gender groups, providing a basis for subsequent multigroup structural analyses. The detailed results of the gender measurement invariance tests are reported in Supplementary Table S3.

**Figure 2:**
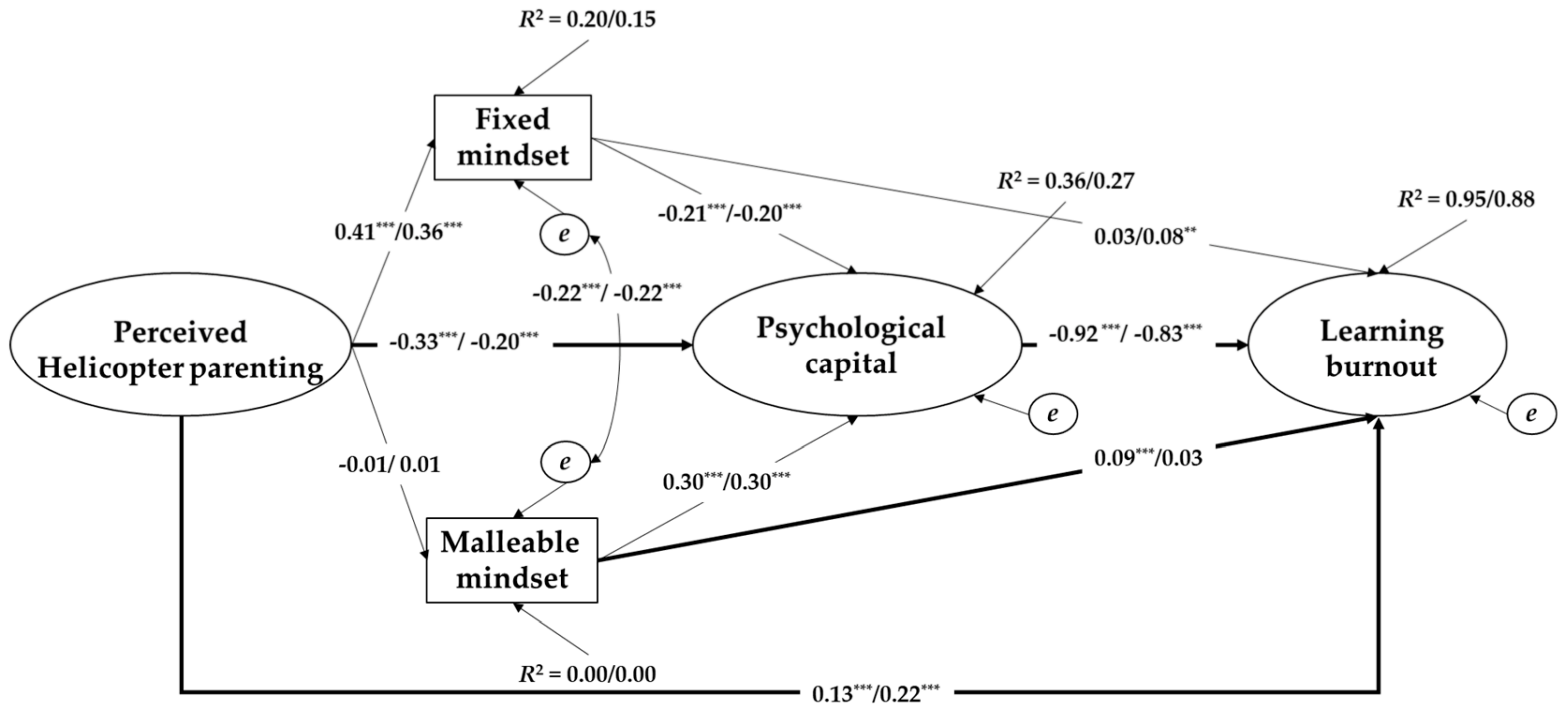
Standardized path coefficients in the Mediation models across gender. Note. Standardized coefficients are presented. Control variables and factor loadings are omitted from the figure for clarity. Two coefficients on the same path are for girls and boys, respectively. Paths in bold indicate the significant gender difference. ** *p* < 0.01, *** *p* < 0.001.

We then examined the path differences across the two groups. As shown in Figure 2, some gender-specific patterns were observed in the mindset–burnout paths. Fixed mindset was positively associated with learning burnout among boys (*β* = 0.08, *p* = 0.001) but not among girls (*β* = 0.03, *p* = 0.176). In contrast, malleable mindset was positively associated with learning burnout among girls (*β* = 0.09, *p* < 0.001), but this association was not significant among boys (*β* = 0.03, *p* = 0.216). The gender-specific indirect effects are presented in Table 4. In the girls’ sample, two indirect pathways were significant: the pathway through psychological capital and the serial pathway through fixed mindset and psychological capital. In the boys’ sample, these two indirect pathways were also significant; additionally, the indirect pathway through fixed mindset alone was significant. By contrast, the indirect pathways involving malleable mindset were not significant in either group.

We further conducted multigroup SEM to examine whether the structural paths differed between boys and girls. Constraining all focal structural paths to equality across gender resulted in a significantly worse model fit, Δ*χ*^2^(9) = 35.20, *p* < 0.001, suggesting that at least some structural paths differed across gender. Path-specific contrasts were then examined in the freely estimated multigroup model. Specifically, the coefficients of four paths across gender were found significant. The negative association between helicopter parenting and psychological capital was stronger among girls than among boys (Δ*β* = −0.14, Δ*b* = −0.190, *p* < 0.001, 95% CI [−0.286, −0.093]). In contrast, the positive association between helicopter parenting and learning burnout was stronger among boys (Δ*β* = −0.09, Δ*b* = −0.041, *p* = 0.025, 95% CI [−0.077, −0.005]). In addition, the path from malleable mindset to learning burnout was significant among girls but not among boys, and the group difference was significant (Δ*β* = 0.06, Δ*b* = 0.031, *p* = 0.025, 95% CI [0.004, 0.058]). Finally, the negative association between psychological capital and learning burnout was stronger among girls than among boys (Δ*β* = −0.01, Δ*b* = −0.053, *p* = 0.001, 95% CI [−0.085, −0.021]). Only the indirect effect of helicopter parenting on learning burnout through psychological capital was significantly stronger among girls than boys, Δ*b* = 0.086, *p* < 0.001, 95% CI [0.049, 0.123]. The full set of path estimates and indirect effects of group-difference tests is presented in Supplementary Tables S4 and S5.

## 4. Discussion

The present study examined the association between perceived helicopter parenting and learning burnout among Chinese adolescents, with intelligence mindsets and psychological capital specified as potential mediating pathways and adolescent gender examined as a moderator. Overall, the findings were consistent with a theoretically plausible model in which perceived helicopter parenting was associated with learning burnout both directly and indirectly. The significant indirect pathways mainly involved fixed mindset and psychological capital. Fixed mindset contributed primarily through its association with psychological capital, whereas malleable mindset did not serve as a mediator because it was not significantly associated with perceived helicopter parenting. The multigroup results revealed several pathway-specific gender differences.

First, perceived helicopter parenting was positively associated with adolescents’ learning burnout. This finding is consistent with prior work showing that helicopter parenting is associated with poorer academic and psychological adjustment, including lower academic motivation, greater emotional problems, and higher school burnout (Ching et al., 2023b; Gao et al., 2023; Love et al., 2020; Schiffrin & Liss, 2017). From the perspective of self-determination theory, the intrusive and autonomy-limiting features of helicopter parenting may be developmentally mismatched with adolescents’ increasing needs for self-direction, competence, and independent coping (Padilla-Walker & Nelson, 2012; Ryan & Deci, 2000). When parents repeatedly intervene, monitor closely, or solve problems on behalf of their children, adolescents may experience academic demands as more externally controlled and psychologically draining (e.g., Angelini et al., 2026). In the Chinese educational context, helicopter parenting may partly overlap with culturally normative academic involvement (Zong & Hawk, 2022), which is often embedded in high-stakes examination pressure, parental educational investment, filial expectations, and strong family aspirations for academic mobility. It should be noted that the present study measured perceived helicopter parenting, reflecting adolescents’ general perception of parental overinvolvement, overprotection, and intrusive management, rather than academic involvement alone. Future research could distinguish domain-specific forms of helicopter parenting, such as parental overinvolvement in academic learning, daily life management, peer relationships, and educational or career decision-making, and examine whether these domains have different implications for adolescent development.

Second, the findings highlight the value of distinguishing fixed mindset from malleable mindset rather than treating them as simple opposites. Consistent with the view that these two beliefs are related but separable dimensions (Costa & Faria, 2018; Lee & Seo, 2019; Yan & Schuetze, 2023), perceived helicopter parenting was associated with stronger fixed mindset but was not significantly associated with malleable mindset. This pattern suggests that adolescents’ perceptions of parental overinvolvement may be more closely linked to heightened concerns about fixed ability than to a simple reduction in beliefs about malleability. Repeated parental intervention may implicitly communicate that adolescents are not fully capable of managing challenges independently, or that mistakes and difficulties are signs of limited competence. Such experiences may be more readily connected with fixed beliefs about ability, which are often associated with evaluation concerns, helpless responses, and avoidance of challenge (Chiu et al., 2023; Yeager & Dweck, 2012). This finding does not imply that malleable mindset is unimportant for academic adjustment; rather, it suggests that malleability beliefs did not explain the link between perceived helicopter parenting and burnout in the present model. Strengthening malleability beliefs alone may therefore be insufficient if adolescents continue to experience strong concerns about fixed ability and external evaluation.

Third, psychological capital appeared to be the most central proximal resource in the model. This finding is consistent with psychological capital theory, which conceptualizes self-efficacy, hope, resilience, and optimism as positive, state-like resources that support adaptive coping and well-being (Luthans & Youssef-Morgan, 2017), as well as with studies linking psychological capital to students’ academic engagement, perceived achievement, well-being, and burnout (N. Chen et al., 2025; Y. Hu et al., 2025; Song et al., 2025; H. Wang et al., 2023). In the present model, perceived helicopter parenting was directly associated with lower psychological capital, and psychological capital was strongly associated with lower learning burnout. This pattern suggests that adolescents’ positive psychological resources may be a particularly important proximal correlate of learning burnout. The negative association between perceived helicopter parenting and psychological capital may reflect the possibility that adolescents who experience parents as overinvolved have fewer opportunities for autonomous problem solving, manageable failure, and self-directed coping. This interpretation is consistent with broader child and adolescent well-being research showing that positive psychological resources and social support are closely related to youth adjustment (Arslan, 2024; Guo & Li, 2025), and with recent evidence linking parental interference to adolescents’ self-efficacy, school anxiety, engagement, depressive symptoms, and burnout (Angelini et al., 2026). In addition, both fixed and malleable mindsets were related to psychological capital. Adolescents who hold stronger fixed beliefs about ability may be less likely to maintain self-efficacy, hope, resilience, and optimism when facing academic difficulties, whereas malleability beliefs may be associated with more adaptive psychological resource profiles (Zhang et al., 2023; Zhao et al., 2023). Thus, although fixed mindset was involved in significant indirect pathways, the explanatory weight of the model appeared to reside largely in psychological capital as a proximal psychological resource rather than in intelligence mindsets alone.

Notably, the direct effect of perceived helicopter parenting on learning burnout remained significant after accounting for the proposed mediators. This indicates that intelligence mindsets and psychological capital explain an important, but not exhaustive, portion of the association. Other parenting-related mechanisms may operate in parallel, such as increased parent–child conflict, perfectionistic pressure, reduced autonomy, lower academic entitlement or agency, and disengagement driven by reactance against excessive control (Madison et al., 2025; Y. Wang et al., 2025a, 2025b). Moreover, learning burnout itself is a multifaceted phenomenon and may also be shaped by broader academic and psychosocial conditions, including workload, examination pressure, teacher expectations, school climate, peer comparison, sleep problems, anxiety, depression, and socioeconomic stress (Z. C. Liang et al., 2025). These factors were beyond the scope of the present model, but they may interact with perceived helicopter parenting or provide alternative pathways to burnout. Accordingly, the present model should be viewed as a substantial but partial account of the association between perceived helicopter parenting and adolescents’ learning burnout.

The gender findings add an important layer of nuance. Rather than showing that perceived helicopter parenting was uniformly more harmful for one gender, the results indicated several pathway-specific gender differences. The paths from perceived helicopter parenting to psychological capital and from psychological capital to learning burnout were stronger among girls than among boys, and the indirect effect through psychological capital was also stronger among girls. This pattern suggests that psychological capital may be a particularly salient proximal resource in girls’ academic adjustment. However, this should not be interpreted as evidence that girls are generally more vulnerable to helicopter parenting, because the direct association between perceived helicopter parenting and learning burnout was stronger among boys. These mixed findings are broadly consistent with related research showing that gender may shape adolescents’ school burnout, psychological adjustment, and responses to parental involvement or interference in complex ways rather than through a single uniform pattern (Angelini et al., 2026; Salmela-Aro & Tynkkynen, 2012). From gender socialization and social role perspectives, boys and girls may encounter different expectations regarding autonomy, achievement, emotional expression, and coping during adolescence, which may influence how they interpret parental involvement and mobilize psychological resources in response to academic stress (Eagly et al., 2000; Hill & Lynch, 1983). Thus, the present findings suggest that gender may moderate specific psychological pathways linking perceived helicopter parenting with learning burnout, rather than indicating a general gendered susceptibility.

One noteworthy result is that malleable mindset was positively associated with learning burnout among girls, whereas this association was not significant among boys. This finding should be interpreted cautiously. It does not necessarily imply that malleable mindset is maladaptive. Rather, after fixed mindset and psychological capital were simultaneously considered, the residual association between malleable mindset and burnout among girls may reflect a more effort-laden form of academic motivation. In highly demanding academic contexts, believing that improvement depends on sustained effort may coexist with heightened self-imposed pressure to persist and perform, especially when adolescents also face strong parental expectations. Under such conditions, malleability beliefs may not directly buffer burnout and may even accompany greater strain. This interpretation is speculative but consistent with recent critiques suggesting that the meaning and effects of growth mindset may vary across contexts and should not be assumed to be uniformly beneficial in all motivational environments (Yan & Schuetze, 2023). More broadly, the present findings suggest that the adaptive value of malleability beliefs may depend on whether adolescents also possess sufficient psychological resources to translate effort beliefs into healthy persistence rather than exhaustion.

Several limitations should be noted. First, although the proposed mediation model was theoretically grounded, the cross-sectional design precludes conclusions about temporal or causal ordering. The sequence from perceived helicopter parenting to intelligence mindsets, psychological capital, and learning burnout should be interpreted as one theoretically plausible specification rather than as an empirically established developmental process. Alternative directions are also possible. Adolescents with greater self-efficacy, hope, resilience, and optimism may be more likely to develop adaptive beliefs about the malleability of intelligence, whereas adolescents with lower psychological capital may become more vulnerable to fixed beliefs about ability. Similarly, adolescents experiencing learning burnout may perceive parental involvement as more intrusive or controlling. Future longitudinal or experimental studies are needed to clarify the temporal ordering and possible reciprocal associations among these constructs.

Second, all major variables were assessed using adolescents’ self-reports, which may introduce common method bias, social desirability bias, and shared perceptual variance. Although the preliminary Harman’s single-factor test suggested that common method bias was unlikely to fully account for the findings, this procedure cannot rule out the possibility of same-source bias. Future research could use multi-informant and multi-method designs, such as combining adolescent reports with parent reports, teacher ratings, school records, behavioral indicators, or longitudinal diary data, to provide a more comprehensive assessment of parenting experiences, psychological resources, and learning burnout. Third, individual chronological age was not collected in this study. Although grade level provided information regarding participants’ educational stage, it cannot fully represent chronological and developmental differences among adolescents. Future research should include precise age information and investigate whether developmental timing moderates the associations among parenting experiences, psychological resources, and learning burnout.

Fourth, the present study assessed perceived helicopter parenting as adolescents’ general perception of parental overinvolvement and did not distinguish whether such parenting behaviors were mainly attributed to mothers or fathers. This distinction may be important because maternal and paternal parenting may have different associations with adolescents’ academic engagement, burnout, and psychological adjustment (e.g., Q. Zhu et al., 2023). Future studies should assess maternal and paternal helicopter parenting separately and examine whether their associations with adolescents’ intelligence mindsets, psychological capital, and learning burnout differ. Finally, although the study included a relatively large sample, participants were recruited from only three public junior high schools in one Chinese province using a convenience cluster sampling approach. Therefore, the findings may not fully generalize to adolescents from different regions, school contexts, socioeconomic backgrounds, or educational systems. Future studies using probability sampling or more diverse samples across multiple regions and school types are needed to further validate the findings.

## 5. Conclusions

This study contributes to the literature by examining how perceived helicopter parenting is associated with learning burnout among Chinese adolescents through intelligence mindsets and psychological capital. The findings suggest that perceived helicopter parenting is positively associated with learning burnout both directly and indirectly. Fixed mindset, rather than malleable mindset, served as the mindset-related mediator, while psychological capital appeared to be the most central proximal psychological resource in the model. The significant sequential pathway through fixed mindset and psychological capital further indicates that adolescents’ beliefs about ability may be linked to burnout partly through their association with positive psychological resources. The gender-specific analyses revealed several pathway-specific differences, but these findings should be interpreted cautiously and do not support a broad conclusion that one gender is generally more vulnerable to helicopter parenting. Overall, the study highlights the importance of distinguishing different dimensions of intelligence mindsets, emphasizing psychological capital in adolescents’ academic adjustment, and considering the culturally specific meanings of parental overinvolvement in the Chinese educational context.

## Figures and Tables

**Table 1:** Sample information.

	Statistics [*n* (%)]
Gender	
Girls	1548 (45.6%)
Boys	1844 (54.4%)
Grade	
Grade 7	1635 (48.2%)
Grade 8	1757 (51.8%)
Ethnic	
Han	3365 (99.2%)
Only Child	
Yes	466 (13.7%)
Intact family	
Yes	3198 (94.3%)
Fathers’ education attainment	
Senior high school and below	1853 (54.6%)
College and above	1235 (36.4%)
Not clear	304 (9.0%)
Mothers’ education attainment	
Senior high school and below	2028 (59.8%)
College and above	1111 (32.7%)
Not clear	253 (7.5%)
Fathers’ job status	
Employed	1784 (52.6%)
Self-employed	1357 (40.0%)
Unemployed	31 (0.9%)
Not clear	220 (6.5%)
Mothers’ job status	
Employed	1459 (43.0%)
Self-employed	1321 (38.9%)
Unemployed	449 (13.2%)
Not clear	163 (4.8%)

Note. The size of total sample is 3392.

**Table 2:** Descriptive statistics of study variables in general and across gender.

	Total Sample (*N* = 3392)			Gender Differences				
				Mean (*SD*)		Levene’s *F*	*t*	Cohen’s *d*
	Mean (*SD*)	Skewness	Kurtosis	Girls (*n* = 1548)	Boys (*n* = 1844)			
1 PHP	2.43 (1.00)	0.37	−0.65	2.43 (1.00)	2.44 (1.01)	0.23	−0.13	0.00
2 Fixed mindset	2.29 (0.94)	0.46	−0.56	2.33 (0.92)	2.26 (0.96)	2.66	2.18 *	0.08
3 Malleable mindset	3.10 (0.91)	0.12	−0.15	3.05 (0.89)	3.14 (0.92)	6.68 *	−2.91 **	−0.10
4 PC_ self-efficacy	4.27 (1.24)	−0.03	−0.01	4.12 (1.22)	4.40 (1.24)	0.92	−6.46 ***	0.22
5 PC_ resilience	4.62 (1.15)	−0.17	0.06	4.23 (1.20)	4.78 (1.09)	3.27	−8.90 ***	0.31
6 PC_ hope	4.99 (1.23)	−0.24	−0.44	4.94 (1.22)	5.02 (1.23)	0.27	−1.99 *	0.07
7 PC_ optimism	4.91 (1.27)	−0.41	−0.14	4.28 (1.30)	4.98 (1.24)	3.17	−3.46 ***	0.12
8 LB_ exhaustion	2.24 (0.76)	0.26	−0.54	2.31 (0.77)	2.19 (0.75)	0.52	4.51 ***	0.16
9 LB_ academic cynicism	1.96 (0.56)	0.92	0.73	1.97 (0.55)	1.95 (0.56)	0.12	1.23	0.04
10 LB_ low achievement	3.17 (0.60)	0.05	−0.12	3.23 (0.60)	3.12 (0.60)	0.51	5.27 ***	0.18

Note. * *p* < 0.05, ** *p* < 0.01, *** *p* < 0.001. PHP = perceived helicopter parenting; PC = psychological capital; LB = learning burnout.

**Table 3:** Correlation coefficients among study variables across gender.

	1	2	3	4	5	6	7	8	9	10
1 PHP	-	0.36 ***	0.03	−0.13 ***	−0.40 ***	−0.24 ***	−0.17 ***	0.46 ***	0.37 ***	0.25 ***
2 Fixed mindset	0.42 ***	-	−0.20 ***	−0.23 ***	−0.35 ***	−0.32 ***	−0.25 ***	0.32 ***	0.39 ***	0.26 ***
3 Malleable mindset	0.00	−0.20 ***	-	0.30 ***	0.20 ***	0.28 ***	0.30 ***	−0.08 ***	−0.13 ***	−0.25 ***
4 PC_ self-efficacy	−0.28 ***	−0.28 ***	0.29 ***	-	0.46 ***	0.75 ***	0.74 ***	−0.22 ***	−0.40 ***	−0.63 ***
5 PC_ resilience	−0.46 ***	−0.34 ***	0.18 ***	0.57 ***	-	0.48 ***	0.45 ***	−0.55 ***	−0.46 ***	−0.46 ***
6 PC_ hope	−0.36 ***	−0.41 ***	0.28 ***	0.73 ***	0.50 ***	-	0.79 ***	−0.30 ***	−0.53 ***	−0.62 ***
7 PC_ optimism	−0.34 ***	−0.38 ***	0.36 ***	0.73 ***	0.55 ***	0.80 ***	-	−0.25 ***	−0.50 ***	−0.54 ***
8 LB_ exhaustion	0.47 ***	0.33 ***	−0.07 **	−0.38 ***	−0.64 ***	−0.42 ***	−0.41 ***	-	0.50 ***	0.36 ***
9 LB_ academic cynicism	0.43 ***	0.41 ***	−0.13 ***	−0.46 ***	−0.46 ***	−0.60 ***	−0.50 ***	0.53 ***	-	0.53 ***
10 LB_ low achievement	0.34 ***	0.31 ***	−0.23 ***	−0.71 ***	−0.53 ***	−0.66 ***	−0.61 ***	0.47 ***	0.56 ***	-

Note. Correlation coefficients from girls are below the diagonal and those from boys are presented above the diagonal. ** *p* < 0.01, *** *p* < 0.001.

**Table 4:** Unstandardized indirect effects from perceived helicopter parenting to learning burnout.

	Girls (*n* = 1548)				Boys (*n* = 1844)			
	*b*	*p*	LLCI	ULCI	*b*	*p*	LLCI	ULCI
Perceived helicopter parenting → Fixed mindset → Learning burnout	0.007	0.177	−0.003	0.017	0.014	<0.001	0.006	0.023
Perceived helicopter parenting → Malleable mindset → Learning burnout	−0.001	0.635	−0.003	0.002	0.000	0.719	−0.001	0.001
Perceived helicopter parenting → Psychological capital → Learning burnout	0.170	<0.001	0.139	0.201	0.084	<0.001	0.062	0.107
Perceived helicopter parenting → Fixed mindset →Psychological capital → Learning burnout	0.044	<0.001	0.032	0.057	0.030	<0.001	0.021	0.039
Perceived helicopter parenting → Malleable mindset →Psychological capital → Learning burnout	0.002	0.632	−0.006	0.010	−0.001	0.709	−0.007	0.005

Note. LLCI = lower limit of confidence interval; ULCI = upper limit of confidence interval.

## Data Availability

The data that support the findings of this study are available on request from the corresponding author.

## Supplementary Materials

The following supporting information can be downloaded at https://www.mdpi.com/article/10.3390/bs16081351/s1. Table S1: School-Level ICCs for the Main Study Variables. Table S2. Standardized Factor Loadings for the Measurement Model. Table S3. Gender Measurement Invariance Tests. Table S4. Gender-Specific Structural Path Estimates and Path-Difference Tests. Table S5. Gender-Specific Indirect-Effect Difference Tests.
