# Supplementary material for: Perceived Helicopter Parenting and Learning Burnout in Chinese Adolescents: Intelligence Mindsets and Psychological Capital as Mediators
Source: Behav Sci (Basel). 2026 Aug 6;16(8):1351. doi: 10.3390/bs16081351 (PMC13509824; doi:10.3390/bs16081351)
Supplement: Supplementary file 1 [file behavsci-16-01351-s001.zip › behavsci-4433046-supplementary.pdf]

Supplementary Table S1. School-Level ICCs for the Main Study Variables

	ICC
1 Perceived helicopter parenting	0.004
2 Fixed mindset	0.000
3 Malleable mindset	0.069
4 PC_ self-efficacy	0.007
5 PC_ resilience	0.014
6 PC_ hope	0.003
7 PC_ optimism	0.019
8 LB_ exhaustion	0.021
9 LB_ academic cynicism	0.008
10 LB_ low achievement	0.011

Note. PC = psychological capital; LB = learning burnout. ICC = Intraclass correlation coefficient.

Supplementary Table S2. Standardized Factor Loadings for the Measurement Model

Latent variables	Indicators	Factor loadings
	Item 1	0.61
	Item 2	0.82
	Item 3	0.60
	Item 4	0.85
Perceived helicopter parenting	Item 5	0.84
	Item 6	0.73
	Item 7	0.71
	Item 8	0.67
	Item 9	0.86
	Self-efficacy	0.84
Psychological capital	Resilience	0.64
	Hope	0.86
	Optimism	0.79
	Exhaustion	0.54
Learning burnout	Academic cynicism	0.67
	Low achievement	0.80

Supplementary Table S3. Gender Measurement Invariance Tests

Model	χ^2	*df*	CFI	TLI	RMSEA	SRMR
Configural	2259.95	176	0.939	0.917	0.084	0.074
Metric	2338.95	189	0.938	0.921	0.082	0.076
Scalar	2476.41	202	0.934	0.922	0.081	0.078

Supplementary Table S4. Gender-Specific Structural Path Estimates and Path-Difference Tests

	Δb	p	95% CI
Perceived helicopter parenting → Fixed mindset	0.045	0.255	[−0.033, 0.123]
Perceived helicopter parenting → Malleable mindset	−0.025	0.545	[−0.107, 0.057]
Perceived helicopter parenting → Psychological capital	−0.190	< 0.001	[−0.286, −0.093]
Fixed mindset → Psychological capital	−0.031	0.434	[−0.110, 0.047]
Malleable mindset → Psychological capital	0.011	0.769	[−0.063, 0.085]
Perceived helicopter parenting → Learning burnout	−0.041	0.025	[−0.077, −0.005]
Fixed mindset → Learning burnout	−0.018	0.204	[−0.046, 0.010]
Malleable mindset → Learning burnout	0.031	0.025	[0.004, 0.058]
Psychological capital → Learning burnout	−0.053	0.001	[−0.085, −0.021]

Note. CI = confidence interval.

Supplementary Table S5. Gender-Specific Indirect-Effect Difference Tests

	Δ*b*	*p*	95% CI
Perceived helicopter parenting → Fixed mindset → Learning burnout	−.007	.275	[−.020, .006]
Perceived helicopter parenting → Malleable mindset → Learning burnout	−.001	.582	[−.003, .002]
Perceived helicopter parenting → Psychological capital → Learning burnout	.086	< .001	[.049, .123]
Perceived helicopter parenting → Fixed mindset → Psychological capital → Learning burnout	.014	.061	[−.001, .029]
Perceived helicopter parenting → Malleable mindset → Psychological capital → Learning burnout	.003	.544	[−.007, .013]
Total indirect effect	.095	< .001	[.057, .134]

Note. CI = confidence interval.
